# Health technology assessment and innovation: here to help or hinder?

**DOI:** 10.1017/S026646232400059X

**Published:** 2024-10-24

**Authors:** Linda Mundy, Ben Forrest, Li-Ying Huang, Guy Maddern

**Affiliations:** 1 School of Public Health, Faculty of Health and Medical Sciences, University of Adelaide, Adelaide, SA, Australia; 2 Access and Value Development, Intuitive Surgical Asia Pacific, Singapore, Singapore; 3 Division of Health Technology Assessment, Center for Drug Evaluation, Taipei, Taiwan; 4 Discipline of Surgery, University of Adelaide, The Queen Elizabeth Hospital, Adelaide, SA, Australia

**Keywords:** technology assessment, health, decision making, health policy, Asia, health services needs and demand, health care evaluation mechanisms, diffusion of innovation

## Abstract

Innovative health technologies offer much to patients, clinicians, and health systems. Policy makers can, however, be slow to embrace innovation for many reasons, including a less robust body of evidence, perceived high costs, and a fear that once technologies enter the health system, they will be difficult to remove. Health technology funding decisions are usually made after a rigorous health technology assessment (HTA) process, including a cost analysis. However, by focusing on therapeutic value and cost-savings, the traditional HTA framework often fails to capture innovation in the assessment process. How HTA defines, evaluates, and values innovation is currently inconsistent, and it is generally agreed that by explicitly defining innovation would recognize and reward and, in turn, stimulate, encourage, and incentivize future innovation in the system. To foster innovation in health technology, policy needs to be innovative and utilize other HTA tools to inform decision making including horizon scanning, multicriteria decision analysis, and funding mechanisms such as managed agreements and coverage with evidence development. When properly supported and incentivized, and by shifting the focus from cost to investment, innovation in health technology such as genomics, point-of-care testing, and digital health may deliver better patient outcomes. Industry and agency members of the Health Technology Assessment International Asia Policy Forum (APF) met in Taiwan in November 2023 to discuss the potential of HTA to foster innovation, especially in the Asia region. Discussions and presentations during the 2023 APF were informed by a background paper, which forms the basis of this paper.

Each year, industry (pharmaceutical, biotech, and device companies) and HTA agency members of the Health Technology Assessment International (HTAi) Asia Policy Forum (APF) attend a three-day meeting to discuss matters of concern in the Asia region. The objective of the 2023 APF was to promote open and constructive dialogue among delegates around the challenges of health technology innovation – how innovation is defined, funded, recognized, and incentivized, and the role HTA can play on the innovation road. Discussions and presentations during the 2023 APF were informed by a background paper, which forms the basis of this paper (1).

## Introduction

Health technology assessment (HTA) is defined as a multidisciplinary process that uses explicit methods to determine the value of a health technology at different points in its lifecycle. The dimensions of value used in HTA include clinical effectiveness, safety, financial and economic implications, ethical, social, cultural, and legal issues, organizational and environmental aspects, as well as considerations of stakeholder impact (2). Using these dimensions, HTA informs the priority-setting process for the investment in, and access to, healthcare technologies, addressing the trade-off between maximizing health and promoting health equity. This is increasingly important when the demand to invest in new health technologies exceeds the limited resources of health budgets (3).

Traditional HTA tends to favor funding decisions that are based on therapeutic value as a measure of innovation, with economic analyses focusing on cost-savings releasing capacity elsewhere in the system, both of which may undervalue new health interventions (4). How innovation is defined, valued, and captured in HTA is inconsistent, and all too often HTA is viewed as a barrier or impediment, rather than a facilitator, of patient access to innovative technologies. However, by recognizing and rewarding innovation in the priority-setting process, decision makers can foster the development of new healthcare technologies.

### How is innovation defined?

Innovation in healthcare is different to innovation in other sectors, in that it tends to result in end-product benefits, such as improved patient outcomes, but usually at a higher cost, as in the case of many pharmaceuticals (5). However, most “new” health technologies only offer incremental benefits rather than being “game-changing” innovations, which, in the context of the health technology field, remain rare. For example, the introduction of a new systemic protein modulator for the treatment of cystic fibrosis was considered to be a truly innovative technology (6). In contrast, although many first in class medical devices are developed, the medical device industry is characterized by incremental innovation, where device versions have short life cycles and are rapidly replaced by newer generation devices. These incremental improvements make it difficult to identify the tipping point where a new iteration of a device represents a truly breakthrough innovation. In addition, medical devices often need to rely on concomitant innovative advances in the surgical procedure required to implant the device, such as the progression from open to minimally invasive surgery to the use of computer aided-or robotic surgery (7;8). The learning curve associated with surgical procedures also needs to be considered when assessing the innovative value of a new device, especially if the learning curve plateau is not achieved before a new iteration of the device is introduced (7).

### How is innovation assessed?

Assessing how truly innovative nondrug technologies are is also difficult due to differences in the quality and level of the available evidence. Clinical studies for new devices are fit for purpose and designed to meet the needs of regulators. It often builds on existing evidence for the technology class and as such may not be a randomized, blinded controlled trial. Evidence describing new diagnostic tests can also be considered limited due to a reliance on indirect, rather than direct evidence of effect, and, in the case of new biomarker or genetic tests, there is often no current reference standard or comparator (7).

It is generally agreed that by explicitly defining what health technology innovation looks like in health policy would allow for appropriate recognition and reward, which will, in turn, stimulate and incentivize future innovation in the system (7;9). The World Health Organization’s definition of innovation is the development of “*new or improved health policies, systems, products and technologies, and services and delivery methods that improve people’s health, with a special focus on the needs of vulnerable populations*” (10). However, this definition could almost fit any new health technology, and highlights the lack of an agreed, overarching definition of innovation across HTA frameworks.

**Figure 1.**
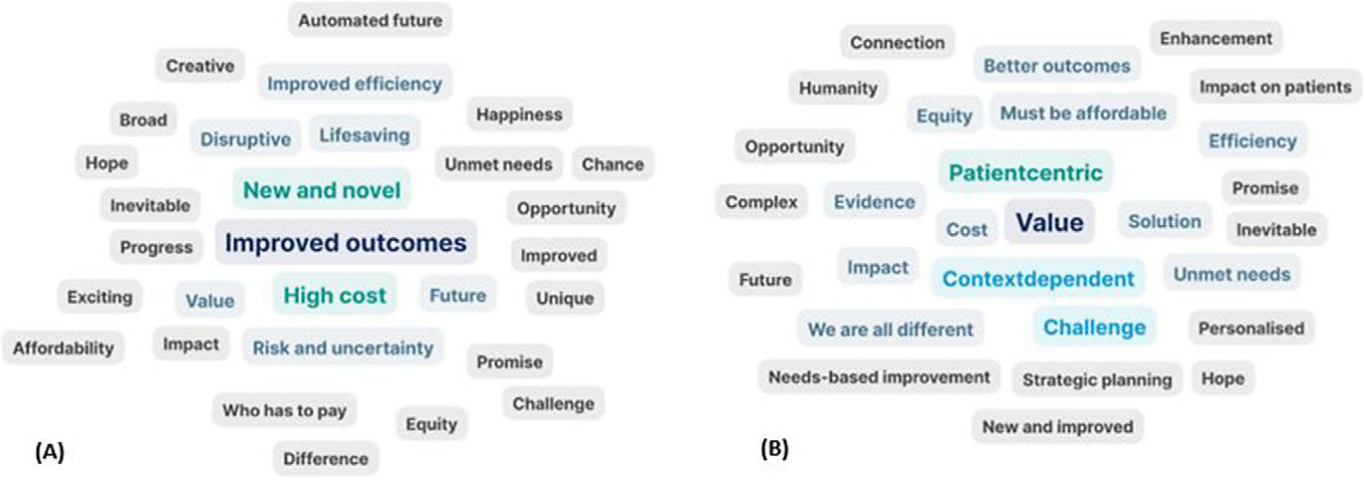
clouds generated by APF delegates, defining innovation on Day 1 (A) and Day 3 (B). APF, Asia Policy Forum.

The varied responses of APF delegates when asked pre- and post-meeting to define what innovation meant to them underlines the lack of a working definition of innovation. There were clear differences in the word clouds generated after 3-days of discussions, with a shift from terms such as “improved outcomes,” “high-cost,” and “new and novel” on Day-1 (Figure 1A) to “value” and “patient-centric” on Day-3 (Figure 1B).

### Policy considerations

Despite wanting to encourage innovation, many health systems do not have any guidance in place to enable policy makers to measure or define innovation, often leaving the decision-making process to the discretion of HTA committee members based on the attributes of the technology in question (5). As such, HTA frameworks do not always state innovation as an *explicit* criterion for the adoption of new health technologies. Innovation is, however, often *implicitly* considered in the priority-setting process, where investment and innovation are encouraged by reducing the financial risk associated with developing new technologies. This is especially true in the evaluation and adoption of new pharmaceuticals where the degree of innovation or novelty is often used by payers in pricing and reimbursement considerations (3;9). Current definitions adopted by payers for innovation in pharmaceuticals tend to focus on therapeutic added value, specifically clinically significant benefits especially when addressing unmet clinical need or severity of disease, health gains (from the patient perspective), and a favorable (from the payer’s perspective) incremental cost-effectiveness ratio (ICER), that is, a risk–benefit ratio at an acceptable cost (5;9).

While policy makers and HTA bodies have struggled to define what constitutes innovation, research has focused on defining a set of criteria that can be applied to gauge whether a new health technology is truly innovative. Two recent systematic reviews identified key papers describing concepts and elements defining innovation, including patient-orientated criteria such as unmet clinical need, added therapeutic value, severity of disease, and public health benefit. Technology-specific criteria were identified, including how disruptive the new technology is (i.e., does the technology replace an existing technology or does it represent only an incremental improvement?), and implementation considerations, including financial and organizational factors (9;11). Similarly, Ciani et al. described three dimensions relating specifically to innovative nondrug health technologies such as medical devices and surgical procedures (see background paper for further details) (1;7). The broad concepts defining innovation for health technologies listed by Rejon-Parrilla were added to, and further distilled by Syeed et al. and are summarized in Table 1 (9–11), noting that many of these attributes overlap each other (e.g., novelty overlaps with substantial benefit and improvement over existing technologies attributes, step-change overlaps with added value).

**Table 1.** Innovation attributes related to healthcare technologies (10;11)

Attribute
*Novelty*: Novelty can address different healthcare demands, including first-in-class drug, a new approach to solve emerging and persistent health issue, or something new/novel to fulfill an unmet need. Broadly, any novel drug, products, services, and clinical practices that introduced over time into the provision of healthcare is a novelty
*Step-change*: Technologies used to treat serious conditions with a potential for significant improvement or lifesaving effects, or unmet clinical need, but at the same time there is high uncertainty around the evidence. Instead of incremental improvement, technologies with breakthrough effect that cure, prevent, or change the course of disease states were considered to offer a step change in terms of outcomes of patients
*Substantial benefit*: Both novelty and step change attributes might potentially overlap with substantial benefit. Technologies that result in a substantial increase in overall survival, substantial contributions to patient safety, large and positive impact on survival, health and quality of life can be considered as providing substantial benefit
*Improvement over existing technologies*: Exhibiting clinically relevant major therapeutic advantages over the existing therapies: improvement in efficacy, effectiveness, safety, bioavailability, and existing products’ quality was referred to as improvement over existing technologies in addition to reducing morbidity in the sick state, increasing longevity, and improving quality of life
*Convenience and/ or adherence*: Optimal route of administration, decreasing the dosage, less frequent dosing (e.g., once daily or once weekly), simplification of multidrug administration along with maintaining appropriate drug release, or improved palatability are all convenience and/or adherence attributes of innovation. In the case of medical devices, portability was recognized as an essential feature of convenience that resulted in widespread acceptance
*Uncounted benefits*: The unnoticed and unrecognized benefits of a technology that produces a demonstrable and distinct benefit that may not have been adequately captured in the technology’s ICER calculation
*Acceptable cost (economic impact)*: Innovation in health care that offers better treatment, better patient care at a reduced cost or cost-neutral compared to existing technologies, that, due to the reduced cost, would improve access to technologies
*Added value*: The technology is expected to alleviate patient/social burden via indirect social benefits with value added to the system through increased productivity and knowledge spillovers

It is likely that the full value of new health technologies will be underestimated if funding decisions do not consider innovation criteria; therefore it would be valuable to develop guidance that *explicitly* states innovation as a criterion in HTA frameworks, rather than innovation being *implicitly* considered by decision makers in the priority-setting process (9;11). Importantly, criteria used to define innovation need to differentiate between therapeutic/clinical or procedural innovation (“therapeutic value”) that results in patient benefit or efficiencies (e.g., novel mode of administration leading to better patient adherence and, as a consequence, greater effectiveness) and commercial or technological innovation that results in a new product with no added value or patient benefit (5;7).

Importantly, healthcare innovation does not have to be complicated or equate to expensive technology, whether that be a medical device or a high-cost pharmaceutical. Innovation can simply be a different way of doing something, a new way of thinking or a simple solution that can be easily applied for the benefit of the intended user. Innovative solutions *may* result in new technology, but innovation does not always equal technology (12).

## HTA mechanisms to promote and stimulate innovation

HTA has several tools at its disposal to enable and foster innovation, including the use of funding mechanisms (e.g., ICER thresholds, managed entry agreements, accelerated access), multicriteria decision analysis (MCDA), and horizon scanning (HS).

### Funding mechanisms

It should be noted that many countries commonly use funding mechanisms in pricing and reimbursement decision making for pharmaceuticals, whereas HTA is used to determine the *value* of a technology at both the individual *and* population levels. Price points ensure affordable and equitable access to new drugs at the same time as providing industry with an incentive to develop new therapies. Pharmaceutical innovation is commonly rewarded by funders agreeing to a premium price for a new drug, widely seen as stimulating innovation while at the same time delivering value for money to the payee. Funders who primarily focus on price minimization may, however, reduce the incentive for future innovation. Again, determining innovation and “real benefit” of a drug is difficult and may consider factors beyond just being a new or novel entity. For example, a new drug that has the same therapeutic effect but can be taken orally at home rather than intravenously in hospital offers real benefit to patients, carers, and healthcare providers. There is a risk; however, for health systems that operate within a limited budget, that by valuing anything beyond therapeutic benefit and inappropriately rewarding “innovation” will displace other, more cost-effective therapies, leading to a decrease in patient outcomes at the population level (5;13). The move toward personalized medicine highlights this issue. Funding high-cost oncology or orphan disease drugs that deliver improved patient outcomes for a relatively few at the individual level may incentivize companies to stop developing incremental improvements in drugs that target common diseases, compromising population-level healthcare (5). This highlights the fine line for decision makers to distinguish between *subsidizing* versus *incentivizing* innovation (14).

*ICER thresholds* represent the maximum value, or threshold, that funders are willing to pay for a health outcome in order to decide whether investing in a new intervention is an efficient use of resources (15). In determining whether new health technologies should be funded, funders prefer explicit to implicit methods of analysis to ensure transparency and consistency of decision making. Most funders have *implicit* ICER values, that is, an “unspoken” ICER value that represents the acceptable price for a unit of additional health gain for which decision makers, on behalf of society, are willing to pay. If the ICER exceeds this threshold value, then technologies will not be funded unless they satisfy other value-based criteria, such as equity or unmet clinical need in rare diseases. Although using an *explicit* ICER threshold is not supported by all funders, applying the same threshold to all technologies and all patient groups does lend a degree of transparency, accountability, and certainty to the funding process (3;6).

Traditional cost-effectiveness analyses are unlikely to capture the full range of benefits of innovative technologies, such as digital health technologies, which may be hugely beneficial but these benefits are hard to capture with a traditional ICER (11). To address this shortcoming, some agencies may justify funding technologies that exceed the ICER threshold by considering ethical and social values such as unmet clinical need or end-of-life treatments, in addition to the innovative nature of the technology (10). The difficulty in defining and capturing innovation in evaluations is reflected in recent changes to NICE’s evaluation guidelines. Previously, NICE explicitly stated that innovation could be used to justify funding technologies that were *not* cost-effective; however, what constituted innovation was not clearly defined and may have resulted in arbitrary funding decisions (3). NICE’s evaluation guidelines now refer to funding decisions for technologies with an ICER above £20,000 per QALY gained, or £100,000 per QALY gained for *highly specialized technologies* that consider “*aspects that relate to uncaptured benefits and non-health factors”* (16).

*Managed entry agreements* (MEAs, also referred to as risk-sharing agreements or patient access schemes), where the risk of investing in high-cost technologies is mitigated by, or shared with, manufacturers. Investment in new healthcare technologies can be challenging for payers in resource-constrained health systems as funding decisions for high-cost, innovative technologies are often associated with a high level of risk and a degree of uncertainty in the (often immature) clinical evidence base, cost-effectiveness, budget impact, price or eligible patient population. With conventional reimbursement decisions, the risk associated with the uncertainty around the true value of a technology is transferred from the manufacturer to the healthcare payer. MEAs share the risk between manufacturers and the payer while at the same time delivering patient access to new technologies (17;18)

MEAs can be classified into two broad categories – health outcomes and financial-based agreements, or sometimes a combination of both; however, the majority of MEAs are financial-based agreements where there is often uncertainties in utilization and budget impact (19). Basic financial agreements include simple discounts agreed between the payer and manufacturer, or price–volume agreements, often applied to drugs with the price of the drug decreasing as more patients receive the treatment (17;20). More complex MEAs are needed to reduce risk and encourage innovation, including value-based pricing, performance-linked reimbursement, and CED, where time-limited funding of technologies is conditional on additional data collection to reduce uncertainties in the (immature) evidence base (18). MEAs allow early patient access to innovative technologies and provide an incentive to manufacturers to produce technologies that are likely to be of value. Outcome-based MEAs, especially for innovative technologies with a limited evidence base such as cell and gene therapies, offer an opportunity to accumulate a robust body of real-world evidence (RWE) to inform future reimbursement decisions as well as recommendations about a technology’s use (14).

Outcome-based MEAs such as CED are not used widely due to the complexity and cost of their implementation including the high cost of administration, data collection and analysis, and an unrealistic definition of value, which is often determined at an early stage of development. Although the use of MEAs has increased over time, they have yet to gain widespread acceptance, mainly due to a lack of evaluation of their effectiveness in meeting their stated goals, that is, increasing patient access and reducing payer risk (17;19;20).

*Accelerated access* is a form of CED where new health technologies receive accelerated regulatory approval by demonstrating early beneficial effects with accumulation of RWE (e.g., progression-free survival) that may translate to real clinical benefits (e.g., overall survival) when post-approval confirmatory studies are completed and evaluated (21). Accelerated access is granted based on limited evidence, and that at the end of the approval period, technologies should undergo HTA evaluation, especially addressing safety, budget impact, and cost-effectiveness (22)

Accelerated access has primarily been used to fast-track the regulatory process predominantly for oncology drugs, expediting access to patients who have little or no treatment options. It does; however, take considerable time for the comprehensive post-marketing evaluation of drugs granted accelerated approval, and as such, the risks and benefits of these drugs remain unclear for some time. A good example of this occurred during the COVID-19 pandemic, where transparent HTA processes were sidelined in favor of emergency use authorizations from regulators operating under the “rule of rescue” for approvals of therapeutics, diagnostics, and vaccines (23). Provisional approvals granted to COVID-19 vaccines were largely successful. However, accelerated approval of many COVID-19 therapeutics, such as emergency authorization given to remdesivir and hydroxychloroquine, were not as successful, with both shown to be either ineffective or harmful after the assessment of real-world data (RWD) (24;25).

Appropriate use of this pathway requires confirmatory trials to be conducted in a timely fashion, using clinically meaningful and validated endpoints, such as overall survival; however, currently, many of these trials are not completed and often it is difficult to remove a drug once it has become available (26;27). Accelerated access could also be used to incentivize innovation and improve healthcare efficiency by providing early patient access to nondrug technologies including new devices, diagnostics, and digital technologies.

### Multicriteria decision analysis

The full range of benefits of innovative technologies may not be captured with the reliance on the use of traditional HTA criteria such as objective comparative measures to determine the approval of new technologies (28;29). The weighting of HTA criteria in the assessment process is often unclear, and innovative technologies may not be funded if evidence of clinical benefit and cost-effectiveness is lacking or insufficient for an investment decision. Decision making can also vary between stakeholders with different perspectives on the benefits of certain medical innovations. These limitations may be overcome with the use of MCDA, a tool that identifies and weighs the attributes of alternative options from multiple stakeholder perspectives. MCDA supports healthcare decision making through the construction of explicit criteria with associated scores or weightings, then ranking, rating, or making pairwise comparisons, ultimately combining multiple factors into a single value (30). As such, criteria can be selected, structured, and then weighted to describe aspects of innovation. For example, Howard et al. defined innovation as a “novel technology not previously used in health care or a totally new indication (or use) of an existing technology, and gave decision makers three discrete choices for the technology in question:

- not innovative: equivalent technology is available or no significant iteration of existing technology;
- incremental innovation: substantial iteration of existing technology or new indication of existing technology; and
- substantial innovation: new technology” (30).

A recent systematic review of MCDAs identified twenty-two different, context-specific criteria that varied according to whether they were used for priority setting or informing clinical or regulatory decision making. Common criteria used were safety, cost and budget impact, quality of care, health outcomes, feasibility, and acceptability. Innovation was seldom used as a criterion (~10 percent), but when it was used, it was in the context of priority setting (28). The use of MCDA has increased in recent years; however, transparency and quality may currently be lacking in its application in the decision-making process. Standardization of MCDA methodology, reporting and selection of criteria according to the intended context (i.e., clinical or regulatory decision making) would improve transparency and enable comparisons across health systems, although defining the type and number of criteria used will depend on the context that the MCDA is intended to be used for (28). MCDA informs stakeholder preferences for early innovation and may expedite early access to beneficial innovations, with a full HTA to follow with the development of evidence. By explicitly contextualizing the MCDA criteria to address localized needs and priorities, MCDAs may add a level of nuance to funding decision making (29). However, more needs to be done to standardize MCDA frameworks, whether they are designed for specific disease areas or general use (22). See background paper for further details (11).

### Horizon scanning

The role of HS was discussed at length at the 2019 APF as a means of reducing uncertainty, enabling planning to facilitate appropriate adoption of health technologies, and providing a degree of future-proofing for health systems (31). Four members of the APF are active members of the HS network International HealthTechScan (formerly EuroScan International Network): Malaysia, Singapore, Taiwan, and South Korea, using HS to inform on new and emerging, innovative health technologies as well as identifying new uses for existing technologies. HS allows policy makers to anticipate and plan, optimizing investment decision making to ensure the successful adoption and implementation of potentially disruptive technologies (32) such as gene and cellular therapies, and oncological pharmaceuticals (33).

An early assessment capability can also respond to demand signaling by actively identifying the needs and key priorities/challenges of a health service, especially scanning and mapping groups of technologies in a clinical care pathway rather than just single technologies. By providing an early assessment of the evidence for new technologies, HS can also feed into MEAs or accelerated access where technologies appear to be beneficial to patients, but robust evidence is lacking to support its full introduction into the health system.

By accelerating policy development and access to health technologies, HS has the potential to provide the link between research and development, speeding up the time to public reimbursement of health innovations (34).

Many of these approaches emphasize the importance of building links between all steps of health technology development: linking industry research and development to academia and HTA, and finally regulators, policy makers and the health system.

## Can HTA foster innovation?

Discussions and presentations during the 2023 APF highlighted the challenges around the role HTA can play in health technology innovation – how innovation is defined, identified, recognized and valued, and funded and incentivized. Innovation was likened to an iceberg heading toward health systems, with HTA only able to “see” (assess) the tip. Both industry and agency delegates agreed that early and affordable access to innovative therapies for patients and clinicians was key, and that there should be an appropriate, sustainable level of investment in innovative therapies in the context of constrained budgets.

To illustrate the role that HTA can play, and to highlight issues, two APF delegates described the HTA journey to bring an innovative technology to market.

### Case study 1: HeartFlow

Noninvasive coronary CT angiography (CTA) is often used to detect and exclude disease patients suspected of coronary artery disease; however, CTA tends to over-estimate the severity of coronary artery stenosis with only a proportion of identified stenoses causing myocardial ischemia (MI). This results in unnecessary downstream diagnostic testing, invasive cardiac catheterization and angiography, and percutaneous coronary interventions (PCI). Fractional flow reserve (FFR) performed during invasive angiography better assesses the hemodynamic significance of lesions compared to CTA; however, invasive FFR carries a risk of serious complications including bleeding, stroke, and MI. HeartFlow is a technology that applies AI-algorithms and deep machine-learning methods to standard coronary CTA images to create a 3-dimensional model of the coronary arteries. The functional severity of a lesion can then be determined under simulated conditions of hyperemia (35;36). When a HeartFlow test is requested, anonymized CTA data are securely transferred from local imaging systems to a United States (US) processing center and used to create 3D computer models of the coronary arteries, incorporating coronary flow characteristics. Results are sent back to the referring clinician, as of 2023, within 6-hr and integrated into a patient’s electronic health record (37).

HeartFlow was developed in the 1990s, received European CE Mark approval in 2011 and US regulatory approval in 2014; however, reimbursement was not yet approved and there was limited uptake of the technology in the US and other countries. In 2014, NICE began to consider HeartFlow, which, according to the NICE innovation criteria, was a novel technology that offered substantial health benefits to the patient (a “step-change”) with innovative characteristics that were difficult to capture in a cost-effective analysis. The HeartFlow company built a collaborative relationship and dialogue with the agency over a 3-year period, providing RWD and RWE on NICE’s advice about evidentiary requirements. In February 2017, NICE completed an HTA and issued positive guidance for the use of HeartFlow FFRCT for estimating fractional flow reserve from coronary CTA. Soon thereafter HeartFlow was identified as an innovative technology by the UK’s Accelerated Access Collaborative based on limited clinical evidence, under the proviso that RWD would be collected. In 2021, NICE updated its evaluation when RWE demonstrated the diagnostic performance of HeartFlow to be equivalent or superior to other noninvasive testing modalities, and a positive HeartFlow FFRCT result better predicted a composite endpoint of death, nonfatal MI and any revascularization compared to a clinically significant stenosis on CTA. Importantly, HeartFlow led to a reduction in invasive coronary angiography procedures, fewer adverse events, and resulted in cost-savings to the health system (37). Supported by NICE’s initial guidance, in 2017 CMS (Centers for Medicare & Medicaid Services) began to fund HeartFlow procedures with a New Technology Ambulatory Payment Classification (APC). US private payers also began to reimburse HeartFlow procedures. In 2018 Japan began to reimburse the technology and in 2022, following the updated NICE guidance, CMS established national payment. HTA was critical to the advancement of HeartFlow’s technology.

The HeartFlow story is indicative of how difficult it can be for an innovative technology, especially in the medical device field where high-level evidence from RCTs is lacking, to undergo a favorable HTA and get positive approval from decision makers. However, early dialogue and use of HTA tools including accelerated access and the analysis of RWD, HeartFlow’s HTA journey advanced from regulatory approval to widespread reimbursement.

### Case study 2: immune checkpoint inhibitors

As part of a normal functioning immune system, the role of immune checkpoint proteins is keeping the immune response of T cells in check. Some checkpoint proteins activate T cells, whilst others switch off the T cells, preventing the destruction of healthy cells. Some cancers express high levels of “switch off” checkpoint proteins, which prevent the T cells from recognizing and destroying cancer cells. Immune checkpoint inhibitors (ICI) work by blocking the cancer’s “switch off” proteins, allowing the T cells to recognize and attack the cancer cells (38).

With uncertain clinical benefits that could result in financial risk and significant budget impact for public funders, these high-cost, innovative treatments are difficult to fund under universal health coverage. However, in response to clinician and patient demand, Taiwan’s single-payer health insurance system, the National Health Insurance Administration (NHIA), agreed to reimburse several ICIs for the treatment of eight cancers. Funding was agreed to under a risk-sharing agreement that required the collection of RWD. For funding to continue, a positive response to ICI treatment in terms of patient-relevant outcomes such as adverse events, progression-free, and overall survival would need to be demonstrated. Data submitted to the NHIA by clinicians included patient baseline and disease characteristics, biomarker profiles, previous surgical and medication histories, and, importantly, patient outcomes over the treatment period (2-year maximum) (39).

Analysis of RWD from 1,644 patients who received ICIs during the 18-month data collection period revealed differences in the effectiveness of ICIs for cancer patients in Taiwan compared to other countries. Outcomes for patients treated with ICIs for urothelial and renal cell carcinoma were found to be better than previously reported by clinical trials. Conversely, based on RWD the NHIA suspended payments for ICIs to treat new cases of gastric and liver cancer, enabling access to other patients who would benefit from the therapy.

By addressing uncertainties in the evidence base, risk-sharing agreements and RWD allows HTA to re-evaluate prior decisions, enabling policy makers to make interim decisions that may facilitate earlier patient access to innovative treatments. HTA and RWE are tools that allow funders and industry to work together to maximize patient benefits and support sustainable healthcare (39), especially early industry involvement in data collection and analysis for reimbursement decision making.

In summary, can HTA support and foster innovation? The HTA community needs to work collaboratively with industry to develop a working definition or value framework of innovation that promotes transparency, accountability, and consistency in the decision-making process. This framework must encompass all medical technologies, not just pharmaceuticals, and especially the new digital and genomic technologies and cellular therapies. Part of this framework may be the inclusion of MCDAs and the development of criteria that can measure value in a meaningful way. Policy makers need to embrace alternative funding mechanisms such as MEAs and CED, again, not just for pharmaceuticals, in so doing mitigating risk for defined periods of time while encouraging the collection and evaluation of RWD. Critically, early dialogue with industry should be promoted, which could be achieved with the establishment of HS and early assessment to understand the potential impact and future value of a technology.
